# Functional UV Blocking and Superhydrophobic Coatings Based on Functionalized CeO_2_ and Al_2_O_3_ Nanoparticles in a Polyurethane Nanocomposite

**DOI:** 10.3390/polym16192705

**Published:** 2024-09-25

**Authors:** Miguel Angel Velasco-Soto, Arturo Román Vázquez-Velázquez, Sergio Alfonso Pérez-García, Lilia Magdalena Bautista-Carrillo, Pavel Vorobiev, Abraham Méndez-Reséndiz, Liliana Licea-Jiménez

**Affiliations:** 1 Centro de Investigación en Materiales Avanzados S.C., Unidad Monterrey, Alianza Norte No. 202, PIIT, Apodaca CP 66628, N.L., Mexico; miguel.velasco85@tec.mx (M.A.V.-S.); arvazvel@gmail.com (A.R.V.-V.); lilia.bautista@cimav.edu.mx (L.M.B.-C.); pavel.vorobiev@cimav.edu.mx (P.V.); abraham.mendez@cimav.edu.mx (A.M.-R.); 2 Instituto Tecnológico y de Estudios Superiores de Monterrey, Eugenio Garza Sada Ave. 2501, Tecnológico, Monterrey CP 64849, N.L., Mexico

**Keywords:** CeO_2_ nanoparticles, Al_2_O_3_ nanoparticles, functionalization, hydrophobic coating, UV protection, polymer nanocomposite

## Abstract

Water repellency has significant potential in applications like self-cleaning coatings, anti-staining textiles, and electronics. This study introduces a novel nanocomposite system incorporating functionalized Al_2_O_3_ and CeO_2_ nanoparticles within a polyurethane matrix to achieve hydrophobic and UV-blocking properties. The nanoparticles were functionalized using an octadecyl phosphonic acid solution and characterized by FTIR and XPS, confirming non-covalent functionalization. Spin-coated polyurethane coatings with functionalized and non-functionalized Al_2_O_3_, CeO_2,_ and binary Al_2_O_3_-CeO_2_ nanoparticles were analyzed. The three-layered Al_2_O_3_-CeO_2_-ODPA binary system achieved a contact angle of 166.4° and 85% transmittance in the visible range. Incorporating this binary functionalized system into a 0.4% *w*/*v* polyurethane solution resulted in a nanocomposite with 75% visible transmittance, 60% at 365 nm UV, and a 147.7° contact angle after three layers. These findings suggest that ODPA-functionalized nanoparticles, when combined with a polymer matrix, offer a promising approach to developing advanced hydrophobic and UV-protective coatings with potential applications across various industrial sectors.

## 1. Introduction

The demand for multifunctional coatings is progressively increasing, driven by various applications in electronics, textiles, self-cleaning coatings, and optical lenses. These coatings require properties such as superhydrophobicity for self-cleaning surfaces and UV-blocking to protect sensitive materials or components from moisture and UV exposure. The engineering of coatings is crucial to achieving these properties. One effective method involves surface modification via nanostructured materials [1], including using long alkyl chains [2], derived from thiols [3], amines [4], phosphonic acid [5], and even through microencapsulation [6]. The most well-known mechanism consists of the interaction and bonding of the anion group to the surface of metal oxides, while the alkyl chain imparts hydrophobicity to the system [7]. Most methodologies utilize the coverage of metallic smooth surfaces with hydroxyl groups to create a functionalized hydrophobic layer. These layers typically achieve static contact angles in the range of 110°–120° [5]. The formation of hydrophobic groups on metallic oxide surfaces often requires prior modification of the nanoparticle’s surface to bond the saturated long chain [8,9], although some prefer an in situ approach [10,11,12]. However, the potential of non-modified metallic oxide nanoparticles remains unexplored. As seen in previous work, there is significant potential in modifying commercially available nanoparticles without pre-modification to impart the desired functionalization [13]. Alumina (Al_2_O_3_) and ceria (CeO_2_) are metal oxide nanoparticles known for their UV radiation absorption capabilities and their use in coatings that, after functionalization, can provide superhydrophobic surfaces [14,15]. The reported contact angles for the bare nanoparticles are ~77° and ~89° for Al_2_O_3_ and CeO_2_, respectively [16]. Functionalized Al_2_O_3_ nanocomposites using a polymeric matrix have been reported to exhibit superhydrophobic behavior with values as high as 158° and 171° [14,17], making them excellent candidates for water-repelling and self-cleaning coatings. Conversely, the functionalization of CeO_2_ has been reported to improve the hydrophobicity of ceria nanocomposites, with contact angles ranging from 95° to 128° [15,18,19,20,21]. Although these values are lower than those obtained for Al_2_O_3_ nanocomposites, CeO_2_ provides UV-blocking characteristics that can be beneficial for extending the lifespan and functionality of coatings. However, ceria films have the disadvantage of high visible radiation absorbance [22], limiting their feasibility for transparent applications.

In contrast, alumina is a low-cost material with high visible transmittance but low UV absorption [23]. Combining both materials could create a synergistic effect in a polymeric matrix with UV-blocking characteristics, making them excellent candidates for UV protection. CeO_2_ polymer nanocomposites have been studied for pyrolytic resistance [24] and UV resistance enhancement [25]. Similarly, Al_2_O_3_ polymer nanocomposites have been researched for improved thermal [26] and electrical conductivity [27]. However, to our knowledge, a combination of both modified nanoparticles for use as hydrophobic and UV-blocking systems has not been studied. Combined, these oxides can create coatings with enhanced hydrophobicity, transparency, and UV resistance, offering improved protection and performance. However, successfully integrating the nanoparticles into a polymer matrix to obtain a nanocomposite with the desired properties requires careful processing and surface modification of the nanoparticle.

In this work, we investigate commercially available nanoparticles with surface modification with octadecylphosphonic acid (ODPA) of individual Al_2_O_3_ and CeO_2_, and a binary composition of both nanoparticles, and the incorporation of the binary system Al_2_O_3_-CeO_2_-ODPA into a polyurethane matrix to develop a hydrophobic nanocomposite. This study addresses the gap in combining these nanoparticles to enhance hydrophobic and UV-blocking properties. The practical implications of this research could significantly impact the development of advanced coatings that can be applied in a wide range of industrial contexts, from electronics to architecture, offering both durability and multifunctionality.

## 2. Materials and Methods

Al_2_O_3_ nanoparticles (Alu C) with a particle size of about 20 nm, a density of 50 g/L, and a specific surface of 85–115 m^2^/g, and CeO_2_ (VP AdNano 90) with a nanoparticle size of 90 nm, a density of 6.13 g/L, and a specific surface of 66 m^2^/g were provided by Evonik, Guadalupe, N.L., México. Octadecylphosphonic acid (ODPA) 97% of chemical grade was purchased from Sigma-Aldrich, and ethanol was purchased from Desarrollo de Especialidades Químicas, Parque Industrial Ciudad Mitras, N.L., México (DEQ). The reagents were used as received. The base and catalyst for the polymer were from the BASF^®^ brand, DC-92 urethane, and DH-50 catalyst.

### 2.1. Nanoparticle Functionalization

#### 2.1.1. Al_2_O_3_ Nanoparticles Functionalized with ODPA

For the preparation of Al_2_O_3_ functionalized nanoparticles, 20 mL of a solution of ODPA 4 mM was added to 250 mg of Al_2_O_3_ nanoparticles and sonicated for 50 min using a pulsed mode, with one second of ultrasound followed by one second of silence. The system was then centrifuged at 4500 rpm for 1 h, and the supernatant was discarded. Subsequently, 10 mL of ethanol was added for washing, followed by 5 min of sonication in an ultrasonic bath. The mixture was centrifuged again under the same conditions (4500 rpm for 1 h). After discarding the supernatant, the solid residue was left to dry at room temperature overnight. Finally, the dried solid was ground into a fine powder using an agate mortar.

#### 2.1.2. CeO_2_ Nanoparticles Functionalized with ODPA

For the preparation of ceria functionalized nanoparticles, 20 mL of an ODPA 4 mM solution was added to a glass vial containing 250 mg of CeO_2_. The mixture was sonicated for 50 min using a pulsed mode, with one second of ultrasound followed by one second of silence. The system was then centrifuged at 4500 rpm for 1 h, and the supernatant was discarded. Subsequently, 10 mL of ethanol was added for washing, followed by 5 min of sonication in an ultrasonic bath. The mixture was centrifuged again under the same conditions (4500 rpm for 1 h). After discarding the supernatant, the solid residue was left to dry at room temperature overnight. Finally, the dried solid was ground into a fine powder using an agate mortar.

#### 2.1.3. Al_2_O_3_-CeO_2_ Nanoparticles Functionalized with ODPA

For the preparation of alumina-ceria functionalized nanoparticles, 20 mL of a 4 mM ODPA solution was added to a glass vial containing 125 mg of CeO_2_ and 125 mg of Al_2_O_3_ nanoparticles. The mixture was sonicated for 50 min using a pulsed mode, with one second of ultrasound followed by one second of silence. The system was then centrifuged at 4500 rpm for 1 h, and the supernatant was discarded. Subsequently, 10 mL of ethanol was added for washing, followed by 5 min of sonication in an ultrasonic bath. The mixture was centrifuged again under the same conditions of 4500 rpm for 1 h. After discarding the supernatant, the solid residue was left to dry at room temperature overnight. Finally, the dried solid was ground into a fine powder using an agate mortar.

#### 2.1.4. Preparation of Al_2_O_3_-CeO_2_-ODPA/Polyurethane Nanocomposite

First, a dispersion was formed with 25 mg of the modified Al_2_O_3_-ODPA, CeO_2_-ODPA, or Al_2_O_3_-CeO_2_-ODPA binary nanoparticles in 4.98 mL of a solvent mixture composed of 75%w ethyl acetate and 25%w toluene. This dispersion was prepared using a sonication tip, at 50% of pulsation for 12 min. Subsequently, 10 μL of DC-92 polyurethane base and 10 μL of DH-46 catalyst were added to the dispersion, followed by stirring with vortex motion for 1 min.

#### 2.1.5. Spin Coating of Nanocomposite Coatings

Borosilicate glass squares (2.5 × 2.5 cm^2^) were cleaned using Citranox detergent, scrubbed with a microfiber cloth, and rinsed with distilled water, followed by deionized water and ethanol. Finally, samples were air-dried and wiped with Kimwipes damped with ethanol. The coatings were deposited onto clean glass using 120 μL of nanoparticle dispersion at a concentration of 0.5% *w*/*v*. The deposition was performed at 1500 rpm for 9 s and 1700 rpm for 20 s, then thermal annealing for 5 min at 100 °C. This process resulted in coatings of Al_2_O_3_, CeO_2_, Al_2_O_3_-CeO_2_, Al_2_O_3_-ODPA, CeO_2_-ODPA, Al_2_O_3_-CeO_2_-ODPA, and Al_2_O_3_-CeO_2_-ODPA/PU, in both single and three-layered configurations. Additionally, the nanocomposite coatings were left to cure for 24 h. To evaluate the transmittance in the UV region, another coating was applied to 2.5 × 2.5 cm^2^ quartz microscope slide substrates (Alfa Aesar, Monterrey, N.L., México).

### 2.2. Characterization

The functionalized nanoparticles were characterized using a Thermo Scientific Nicolet iS50 FT-IR by FTIR in ATR mode, with measurements taken in air from 4000 to 400 cm^−1^. XPS analysis was performed using a Thermo Scientific EscaLab 250Xi Instrument, East Grinstead, UK with monochromated Al Ka (1486.6 eV) X-ray source, generated at 14 kV, a base pressure of 10^−10^ mbar, with a 650 μm spot, a pass energy of 20 eV, and an energy step size of 0.100 eV. The nanoparticles and nanocomposite coatings were analyzed using UV-vis spectroscopy in transmittance mode with a VARIAN Cary 5000 UV-vis-NIR spectrophotometer, in the range of 200–800 nm in air. The mean contact angle was measured using a Dataphysics contact angle system OCA 15plus, employing a 60 μL water droplet with the static sessile drop method, analyzing eight zones of room temperature in air, following the ASTM5725-99 standard, with SCA20 software used for the analyses. Finally, the nanocomposite was analyzed with an atomic force microscope in tapping mode using an Asylum Research MFP3D-SA, with an AC240TS-R3 rectangular cantilever, resonance at 70 kHz, and a spring constant of 2 N/m.

## 3. Results and Discussion

### 3.1. Al_2_O_3_, CeO_2_, and Al_2_O_3_-CeO_2_ Nanoparticle Functionalization

**Figure 1:**
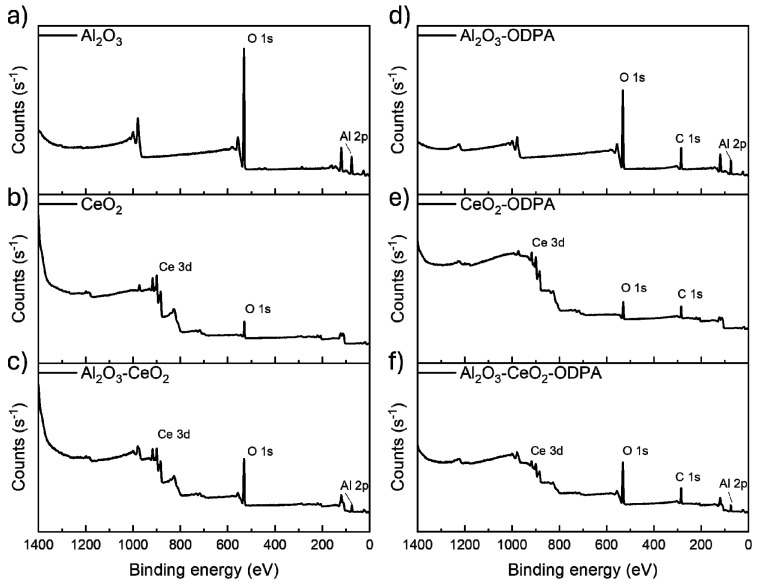
XPS survey spectra for unmodified and modified nanoparticles where (**a**–**c**) show the spectra of the single and binary nanoparticles without functionalization and (**d**–**f**) show the single and binary nanoparticles after functionalization with ODPA, showing the presence of C 1s signal due to the presence of the organic molecule.

The XPS survey analysis of nanoparticles, as shown in Figure 1a–c, presents the spectra for the non-modified oxide nanoparticles, where the main signals present are attributed to the metals (Al 2p, Ce 3d) and oxygen (O 1s), with no indication of trace impurities. After treatment with ODPA, Figure 1d–f show survey spectra of the functionalized nanoparticles, which display the same signals from the metal oxides and a new signal corresponding to C 1s, confirming the presence of the ODPA molecule. To further characterize the nanoparticle’s functionalization, XPS high-resolution spectra were obtained. This analysis provided atomic concentrations (see SI Table S1). For Al_2_O_3_ and CeO_2_, the ratio between metal and oxygen was nearly stoichiometric, confirming the purity of the nanoparticles. Additionally, adventitious carbon was present in the non-modified nanoparticles. In the functionalized nanoparticles, the carbon concentration increased, accompanied by the emergence of a phosphorus signal, which further confirmed the presence of ODPA.

**Figure 2:**
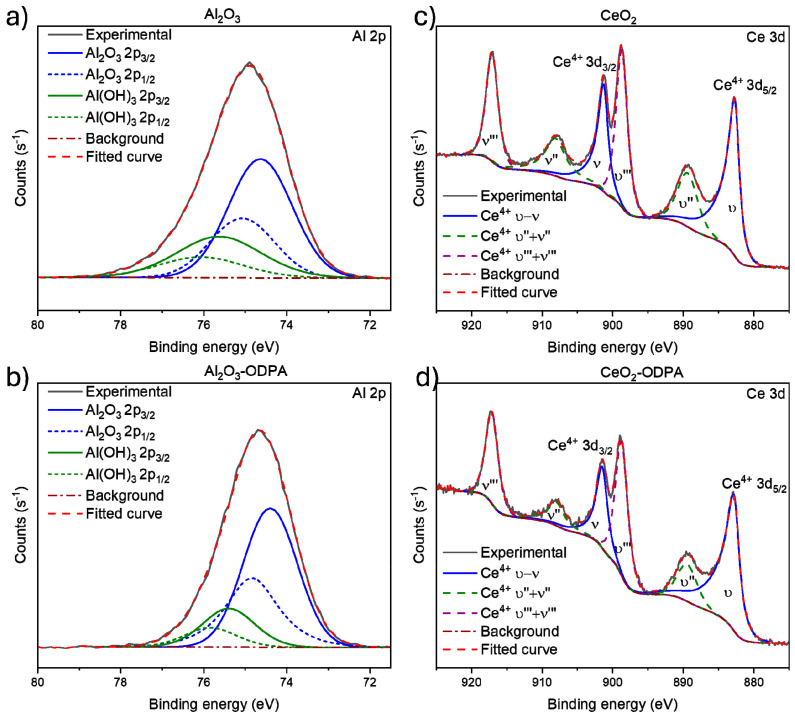
XPS high-resolution spectra for the Al 2p region for (**a**) Al_2_O_3_, (**b**) functionalized Al_2_O_3_, (**c**) CeO_2_, and (**d**) functionalized CeO_2_.

Figure 2a presents the high-resolution spectra for Al 2p region, showing a doublet signal at 74.63 eV and 75.07 eV that can be assigned to Al 2p_3/2_ and Al 2p_1/2_, respectively, originating from Al_2_O_3_ [28]. Additionally, there is a doublet associated with aluminum bonded to hydroxyl groups. After functionalization (Figure 2b), Al 2p region exhibits the same signals as the non-modified nanoparticles with a slight shift of around 0.2 eV in the binding energy of the aluminum doublet signals. This shift has been reported to be caused by organic molecules like ODPA being electrostatically adsorbed on the nanoparticle’s surface, suggesting that the functionalization is non-covalent [29,30,31]. For CeO_2_, Figure 2c displays the high-resolution spectra in the Ce 3d region where six doublets (υ+ν, υ″+ν″, and υ‴+ν‴) were used, with a doublet signal at 882.70 eV and 901.3 eV corresponding to Ce^4+^ 3d_5/2_ and 3d_3/2_, respectively, with an orbital splitting of 18.6 eV [32,33]. Additional signals correspond to Ce^4+^ states typically associated with cerium oxides [33]. The asymmetry in the different signals might arise from a contribution from Ce^3+^ states. Nevertheless, the concentration is minimal since no appreciable shoulders are observed, and the Ce-O ratio is nearly stoichiometric for CeO_2_ [33]. In Figure 2d, the spectra for the CeO_2_ functionalized nanoparticles show roughly identical signals, with a slight shift in position (0.1–0.2 eV) similar to that observed in Al_2_O_3_-ODPA spectra. This behavior suggests that the functionalization is also non-covalent, involving electrostatic interaction. Similar behavior was observed in the alumina-ceria nanoparticles (see SI Figures S1 and S2).

**Figure 3:**
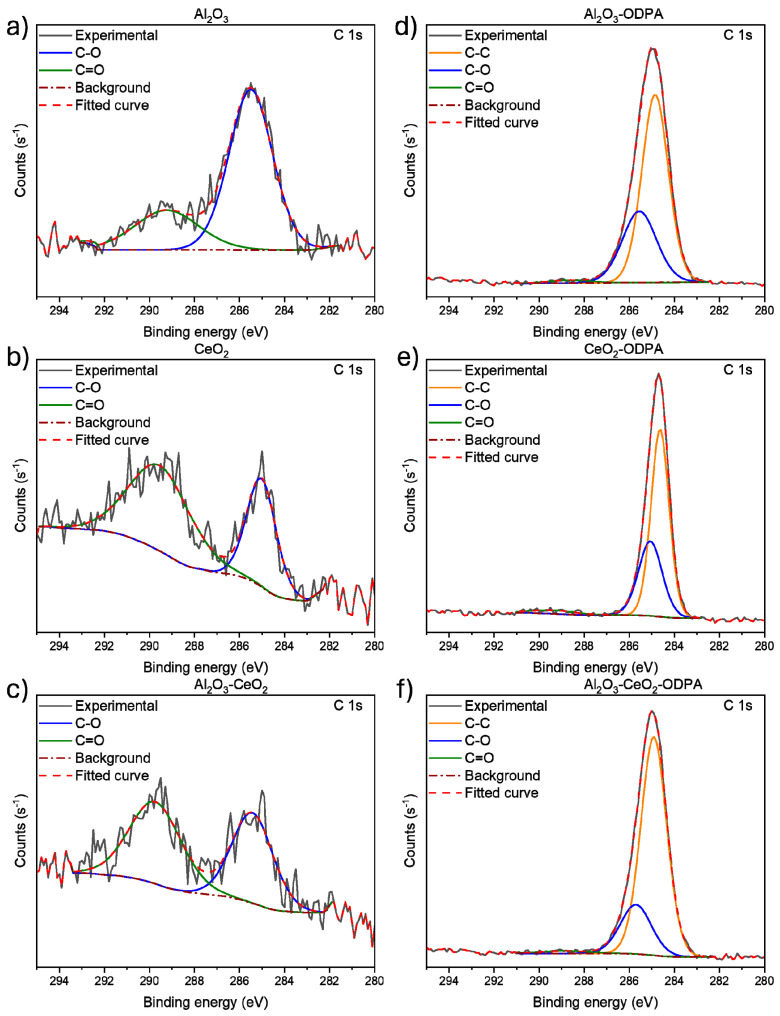
XPS high-resolution spectra for the C 1s region for (**a**) Al_2_O_3_, (**b**) CeO_2_, (**c**) Al_2_O_3_-CeO_2_, (**d**) Al_2_O_3_-ODPA, (**e**) CeO_2_-ODPA, and (**f**) Al_2_O_3_-CeO_2_-ODPA.

XPS high-resolution spectra for the C 1s region for all the nanoparticles are shown in Figure 3. Here, for the non-modified nanoparticles (Figure 3a–c), the carbon region exhibits weak signals corresponding to adventitious carbon. After functionalization (Figure 3d–f), a signal corresponding to C-C, due to the alkyl chain in ODPA, is observed, with no other significant signals. This finding supports the presence of functionalization via non-covalent interaction. Similar observations are made in the oxygen spectra for the different nanoparticles (see SI Figure S3).

**Figure 4:**
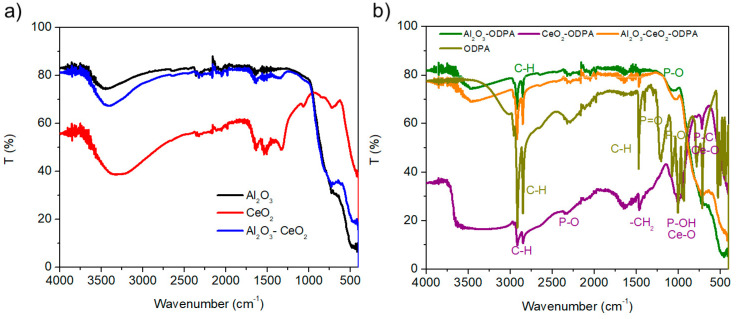
FTIR spectra for (**a**) unmodified Al_2_O_3_, CeO_2,_ and Al_2_O_3_-CeO_2_ and (**b**) modified Al_2_O_3_, CeO_2,_ and Al_2_O_3_-CeO_2_. compared against ODPA.

Further analysis using IR spectroscopy shows the spectra for non-modified nanoparticles in Figure 4a and functionalized nanoparticles Figure 4b, with the isolated ODPA spectrum also included for comparison. The Figure shows that after modification, C-H stretching signals are present from the 2800–2700 cm^−1^ region, also P-O bonding near the 1000 cm^−1^ region; modified CeO_2_ versus ODPA, in which signals of C-H bonding are present and modified Al_2_O_3_-CeO_2_ versus ODPA. The analysis suggests that the initial nanoparticles have no adsorbed organic materials, as indicated by the absence of absorption in organic regions. In contrast, the modified nanoparticles display broad signals around 1000 cm^−1^, characteristic of P-O groups that are surface-bonded via electrostatic interactions [2]. This indicates successful surface modification of the nanoparticles, as suggested by the XPS analysis. Notably, CeO_2_ appears to exhibit the strongest interaction with ODPA, which is corroborated by the XPS analysis showing a higher percentage of phosphorus in the CeO_2_-ODPA nanoparticles (refer to SI Table S1).

Phosphonic acids are well-known as effective ligands, exhibiting various bonding modalities to surface hydroxyl groups. It is generally accepted that bonding to ion species with a high valence charge produces low-water-soluble compounds [7]. Additionally, it is known that elevated temperatures, around 100 °C, promote the formation of M-O-P bonds. FTIR spectra show that phosphonic acids are deprotonated for the Al_2_O_3_-ODPA, suggesting that a condensation reaction may occur between the phosphonic acid and Al_2_O_3_ surfaces. In the case of CeO_2_-ODPA, the intensity of the P=O bonding diminishes, indicating that the P=O group is reacting with the CeO_2_ surface. Moreover, we hypothesize that ultrasonication not only aids in deagglomerating the metal oxide nanoparticles but also provides the necessary energy to accelerate the formation of these bonds. As reported in other studies, when the ODPA solution is left to react without external energy sources, a saturation coverage period of approximately 24 h is required [5]. Therefore, the method presented here could offer a rapid approach to functionalize metal oxide nanoparticles, potentially reducing the time required for surface coverage by about 2500%.

### 3.2. Nanoparticles and Nanocomposite Properties

**Figure 5:**
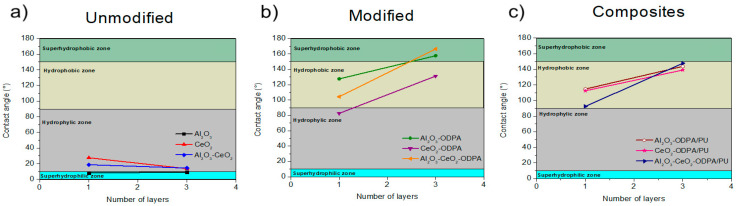
Representation of the variation of the contact angle of (**a**) non-modified, (**b**) functionalized nanoparticles, and (**c**) nanocomposite with the number of deposited layers.

**Figure 6:**
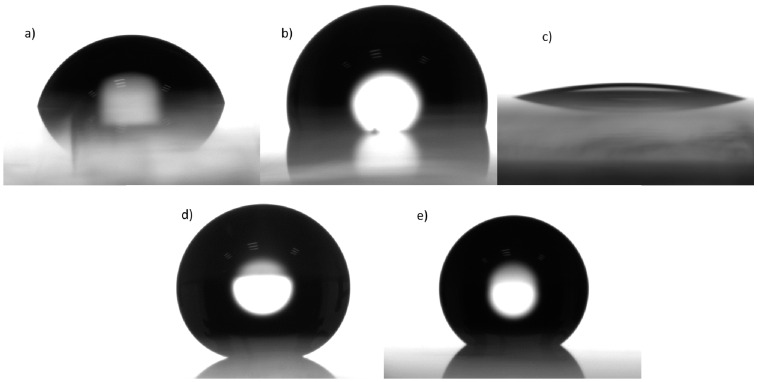
Images of water contact angle for (**a**) polyurethane 79.51° (PU); (**b**) ODPA 106.49°; (**c**) Al_2_O_3_-CeO_2_ 0.5% *w*/*v* 14.45°; (**d**) Al_2_O_3_-CeO_2_-ODPA 0.5% *w*/*v* 166.4°; (**e**) Al_2_O_3_-CeO_2_-ODPA 0.5% *w*/*v*/PU, 147.7°.

The resulting 0.5% *w*/*v* nanoparticle coatings exhibited a wide arrange of contact angles when deposited as one or three layers, as shown in Figure 5. The non-modified nanoparticles in Figure 5a displayed contact angles within the hydrophilic zone, in the range of 10°–90°. Specifically, Al_2_O_3_ obtained 8.13° and 9.2° for one and three layers, respectively, while CeO_2_ exhibited angles of 27.39° and 13.68°. The Al_2_O_3_-CeO_2_ yielded angles of 18.71° and 14.45°, as shown in Figure 5a and Figure 6c. For the functionalized nanoparticles, Al_2_O_3_-ODPA achieved contact angles of 127.52° and 157.76°, while CeO_2_-ODPA showed 82.56° and 131.11°. The modified nanoparticles in Figure 5b produced results ranging from hydrophilic to hydrophobic, reaching the superhydrophobic zone. The binary modified system with a single layer of Al_2_O_3_-CeO_2_-ODPA resulted in a contact angle of 104.25°, while the highest angle was observed for the three-layered Al_2_O_3_-CeO_2_-ODPA system, achieving 166.4°, as depicted in Figure 5b and Figure 6d. Moreover, to determine if ODPA alone could result in superhydrophobicity, a 4 mM solution was spin-coated in a glass substrate, with 1 to 10 layers deposited. The results showed that the surface contact angle with water increased to a maximum of 106.49°, regardless of the number of layers. This indicates that for ODPA, the contact angle was independent of the number of deposited layers, with a maximum saturation angle of 106°, which has been previously reported [34], as shown in Figure 6b. Therefore, it can be partially concluded that the hydrophobicity observed is not solely due to the incorporation of the alkyl phosphonic acid but also the presence of the nanoparticles and the number of deposited layers. Finally, Al_2_O_3_, CeO_2,_ and Al_2_O_3_-CeO_2_-ODPA were selected for incorporation into the polyurethane matrix (PU) matrix, as shown in Figure 5c. The water contact angle of the PU alone was 79.51°, as shown in Figure 6a.

The dispersions of Al_2_O_3_-ODPA 0.5% *w*/*v*/PU, CeO_2_-ODPA 0.5% *w*/*v*/PU, and Al_2_O_3_-CeO_2_-ODPA 0.5% *w*/*v*/PU were also deposited in one and three layers, giving contact angles of 127.52° and 157.76° for the alumina nanocomposite, 82.56° and 131.11° for the ceria nanocomposite, and 90.1° and 147.7° for the alumina-ceria nanocomposite, respectively, as shown in Figure 5c and Figure 6e. These contact angles are consistent with the values obtained for alumina nanocomposites (158°–171°) and are among the highest reported for ceria nanocomposites (95°–128°) [14,15,17], confirming the synergistic effect of the materials. The behavior of increasing water contact angle with the number of layers remains consistent, with results surpassing those of PU or ODPA alone. Additionally, compared to other modified metallic oxide surfaces [5], the aluminum oxide modified surface in this study exceeded the contact angle by 56°. The highest reported hydrophobic angle for CeO_2_ was found to be 163° [35]; however, in that case, sputtering was used to create a textured ceria surface, followed by treatment with KOH to activate the surface. Although the angle from that work surpasses the hydrophobicity of our modified CeO_2_, it is noteworthy that combining CeO_2_ with modified Al_2_O_3_ nanoparticles in this study generated a synergistic effect, achieving similar results without the need for harsh conditions or prolonged treatment times. As far as we know, this is the first time a binary system of commercially available alumina and ceria reported having superhydrophobic properties without requiring such intensive processing.

**Figure 7:**
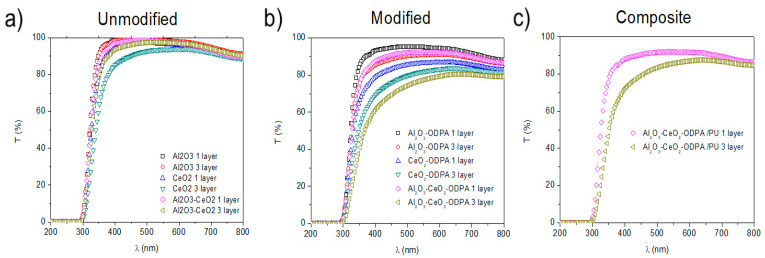
UV-vis transmission spectra for one and three layers of 0.5% *w*/*v* (**a**) unmodified Al_2_O_3_, CeO_2_, and Al_2_O_3_-CeO_2_, (**b**) functionalized Al_2_O_3_, CeO_2_, and Al_2_O_3_-CeO_2_, and (**c**) Al_2_O_3_-CeO_2_-ODPA/PU composites. Non-modified Al_2_O_3_ is independent of the deposited layers, while the modified form encounters a decrease in the transmittance while increasing the layers. The number of layers of non-modified CeO_2_ affects the transmittance. Additionally, the modified ones tend to have higher visible absorption. Non-modified Al_2_O_3_-CeO_2_ shows a predominant behavior of the Al_2_O_3_ transmittance independence while non-modified, while being functionalized, shows the behavior of both combined, with higher visible wavelength absorption. Al_2_O_3_-CeO_2_-ODPA 0.5% *w*/*v* PU nanocomposite shows a higher transmittance in the visible region than the functionalized binary system alone.

The transparency of the unmodified and modified nanoparticles, along with Al_2_O_3_-CeO_2_-ODPA/PU 0.5% nanocomposites in one and three-layer films, was also analyzed in the visible region, as shown in Figure 7. The transmittance of Al_2_O_3_ non-modified films was found to be independent of the number of deposited layers. However, for ODPA-modified versions, transmittance tended to decrease with the number of layers, as shown in Figure 7a. For the CeO_2_ non-modified layers, the transmittances in the visible region decreased with an increasing number of layers, and a similar trend was observed for the surface-modified versions. Notably, strong UV absorption near 350 nm suggests that the organic molecule contributed to this absorption, as shown in Figure 7b. In the case of the binary system Al_2_O_3_-CeO_2_-ODPA, the transmittance behavior appeared to be a combination of both individual nanoparticle systems, as seen in Figure 7b. While the absorption of unmodified nanoparticles showed little dependence on the number of layers, the behavior of Al_2_O_3_ nanoparticles dominated in the unmodified state. After the surface modification, the binary system exhibited characteristics of both CeO_2_ and Al_2_O_3_, with a notable reduction in transmittance in the visible spectrum. The Al_2_O_3_-CeO_2_-ODPA/PU, 0.5% nanocomposite, showed similar behavior to the Al_2_O_3_-CeO_2_-ODPA nanoparticles, but the presence of the polymer appeared to enhance transmittance, possibly due to better nanoparticle dispersion on the glass surface. Transmittance decreased as the number of layers increased (Figure 7c). The three-layer sample had two additional optical interphases compared to the one-layer structure. However, the presence of PU, especially in a three-layer configuration where transmittance noticeably increased compared to PU-free samples, slightly changed the slope near the UV region (Figure 7c versus Figure 7b for comparison). This can be attributed to the fact the optical conductivity and the refractive index of PU increased with the higher photon energy, which occurred near the UV region. It is also known that the optical dispersion curves of PU are well fitted with the Wemple DiDomenico relation, explaining a general increase in transmittance and characterizing PU as a suitable material for use as a window in solar cell applications [36].

**Figure 8:**
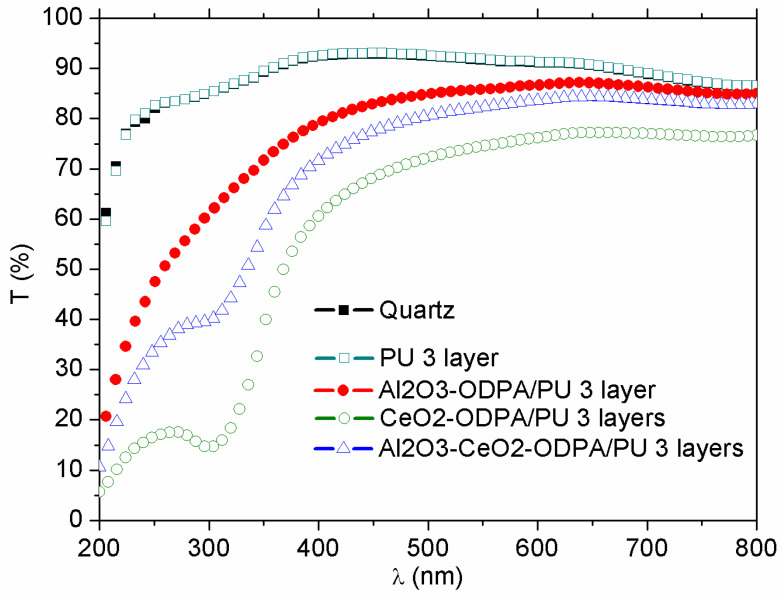
UV-vis transmission spectra for one and three layers of 0.5% *w*/*v* PU nanocomposites cast onto quartz substrates.

To analyze the contribution of the nanocomposites in the UV region, coatings were cast onto quartz substrates, under the same conditions, with only the three-layered coatings being analyzed. Quartz was transparent in the UV region, as shown in Figure 8. The Al_2_O_3_-ODPA 0.5%/PU nanocomposites were transparent across most of the visible region but began to reduce transmittance at 400 nm. The CeO_2_-ODPA 0.5% PU nanocomposite exhibited the highest UV blockage, although it also showed low visible transmittance. The binary system, however, combined the desirable aspect of both qualities, offering a relatively high transmittance in the visible range while effectively blocking UV radiation. This behavior correlates with previous studies on the optical properties of CeO_2_ and Al_2_O_3_ nanoparticles, which report significantly higher absorbance levels for CeO_2_ near the UV region [37,38].

**Figure 9:**
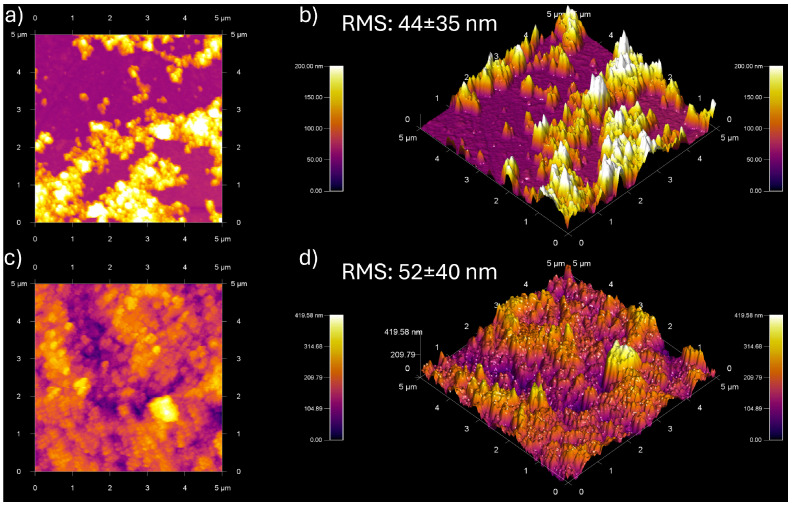
AFM micrography for one- and three-layer nanocomposites based on Al_2_O_3_-CeO_2_-ODPA 0.5% *w*/*v* PU. The one-layer sample (**a**) shows low coverage for the glass, with (**b**) an RMS roughness of 44 nm, indicating that low contact angles are due to the non-homogeneous surface. In contrast, the three-layered nanocomposite (**c**) presents a more homogeneous surface, although (**d**) the RMS roughness is 52 nm. This suggests that the hydrophobic effect has to be a combined effect of roughness, coverage, and the surface modification of the nanoparticles.

AFM micrographs were taken for the one-layered and three-layered nanocomposites to understand further the effect of the number of layers on the water contact angle, as shown in Figure 9. The analysis revealed that the one-layer nanocomposite did not achieve complete coverage by the nanoparticles (Figure 9a), with a roughness of approximately 44 nm (Figure 9b) and a mean layer thickness ranging between 20 nm and 110 nm. In contrast, the three-layer nanocomposite showed homogeneous coverage (Figure 9c), with an increased roughness of 52 nm (Figure 9d) and also a higher standard deviation of 40 nm, indicating the existence of numerous valleys and peaks, in contrast with the one-layer nanocomposite and a thickness range of 150–300 nm. These results suggest that increasing the number of layers and surface roughness contributes to achieving the Cassie-Baxter state, thereby inducing the superhydrophobic effect. This phenomenon has been observed with the addition of more layers [14,17,19].

## 4. Conclusions

In this work, we successfully developed a nanocomposite based on Al_2_O_3_-CeO_2_-ODPA nanoparticles in a 0.5% *w*/*v* concentration within a polyurethane matrix. This is the first time such a nanocomposite has been reported. Our findings indicate that ODPA is an effective alternative for creating hydrophobic coatings. The number of deposited layers significantly influenced the surface roughness and, consequently, the water contact angle, with the highest angle of 166.4° for Al_2_O_3_-CeO_2_-ODPA nanoparticles, which reduced to 147.7° when incorporated into the polyurethane matrix. Despite these promising results, further research is needed to optimize several parameters, including the concentration of polyurethane, the spin coating speed of the spin, and the measurement of adhesion to various substrates. Expanding this research could broaden the applicability and effectiveness of these hydrophobic coatings across multiple industries.

## Data Availability

The original contributions presented in the study are included in the article/Supplementary Material, further inquiries can be directed to the corresponding author/s.

## Supplementary Materials

The following supporting information can be downloaded at https://www.mdpi.com/article/10.3390/polym16192705/s1, Table S1: Atomic composition for the different nanoparticles obtained from XPS high-resolution spectra; Figure S1: XPS high-resolution spectra for the Al 2p region from (a) Al_2_O_3_, (b) Al_2_O_3_-ODPA, (c) Al_2_O_3_-CeO_2_, and (d) Al_2_O_3_-CeO_2_-ODPA. Figure S2: XPS high-resolution spectra for the Ce 3d region from (a) CeO_2_, (b) CeO_2_-ODPA, (c) Al_2_O_3_-CeO_2_, and (d) Al_2_O_3_-CeO_2_-ODPA. Figure S3: XPS high-resolution spectra for the O 1s region from (a) Al_2_O_3_, (b) CeO_2_, (c) Al_2_O_3_-CeO_2_, (d) Al_2_O_3_-ODPA, (e) CeO_2_-ODPA, and (f) Al_2_O_3_-CeO_2_-ODPA.
