# Supplementary material for: Functional UV Blocking and Superhydrophobic Coatings Based on Functionalized CeO2 and Al2O3 Nanoparticles in a Polyurethane Nanocomposite
Source: Polymers (Basel). 2024 Sep 25;16(19):2705. doi: 10.3390/polym16192705 (PMC11478342; doi:10.3390/polym16192705)
Supplement: Supplementary file 1 [file polymers-16-02705-s001.zip › polymers-3192520-supplementary.pdf]

## Supplementary Information (SI)

# FUNCTIONAL UV BLOCKING AND SUPERHYDROPHOBIC COATINGS BASED ON FUNCTIONALIZED $CeO_2$ AND $Al_2O_3$ NANOPARTICLES IN A POLYURETHANE NANOCOMPOSITE

**Miguel Angel Velasco-Soto [1,2], Arturo Román Vázquez-Velázquez [1], Sergio Alfonso Pérez-García[1*],Lilia Magdalena Bautista-Carrillo[1], Pavel Vorobiev[1], Abraham Méndez-Reséndiz[1], and Liliana Licea-Jiménez [1*]**

[1] *Centro de Investigación en Materiales Avanzados S.C., Unidad Monterrey, Alianza Norte No. 202, PIIT, Apodaca, N.L. CP 66628, México*

[2] Instituto Tecnológico y de Estudios Superiores de Monterrey, Eugenio Garza Sada Ave. 2501, Tecnológico, Monterrey, NL, México

* Correspondence: liliana.licea@cimav.edu.mx; alfonso.perez@cimav.edu.mx

### X-ray photoelectron spectroscopy (XPS)

For XPS, samples were analyzed in a Thermo Scientific Escalab 250Xi instrument. During analysis, the base pressure was ~$10^{-10}$ mbar, and the photoelectrons were generated with the AlKα (1486.68 eV) X-ray source using a monochromator and a spot size of 650μm. The X-ray voltage and power were 14 kV and 350 W, respectively. The acquisition conditions for the high-resolution spectra were 20 eV pass energy, 45º takeoff angle, and 0.1 eV/step. The recorded photoelectron peaks were fitted using the Avantage Software V 5.9931. The signals were analyzed using a Gaussian–Lorentzian function and a smart background subtraction. The smart background is a variation of Shirley's background, preventing the background from being above the actual data. Table S1 shows the atomic percentages calculated from the high-resolution XPS spectra.

Table S1. Atomic composition for the different nanoparticles obtained from XPS high-resolution spectra.

| | Al % | O% | Ce% | C % | P % |
|---|---|---|---|---|---|
| $Al_2O_3$ | 39.48 | 56.96 | - | 3.56 | - |
| $CeO_2$ | - | 57.71 | 30.31 | 11.99 | - |
| $Al_2O_3$-$CeO_2$ | 30.34 | 59.52 | 6.21 | 3.94 | - |
| $Al_2O_3$-ODPA | 30.23 | 41.81 | - | 26.24 | 1.72 |
| $CeO_2$-ODPA | - | 32.8 | 10.65 | 52.87 | 3.68 |
| $Al_2O_3$-$CeO_2$-ODPA | 23.25 | 40.24 | 2.89 | 31.55 | 1.82 |

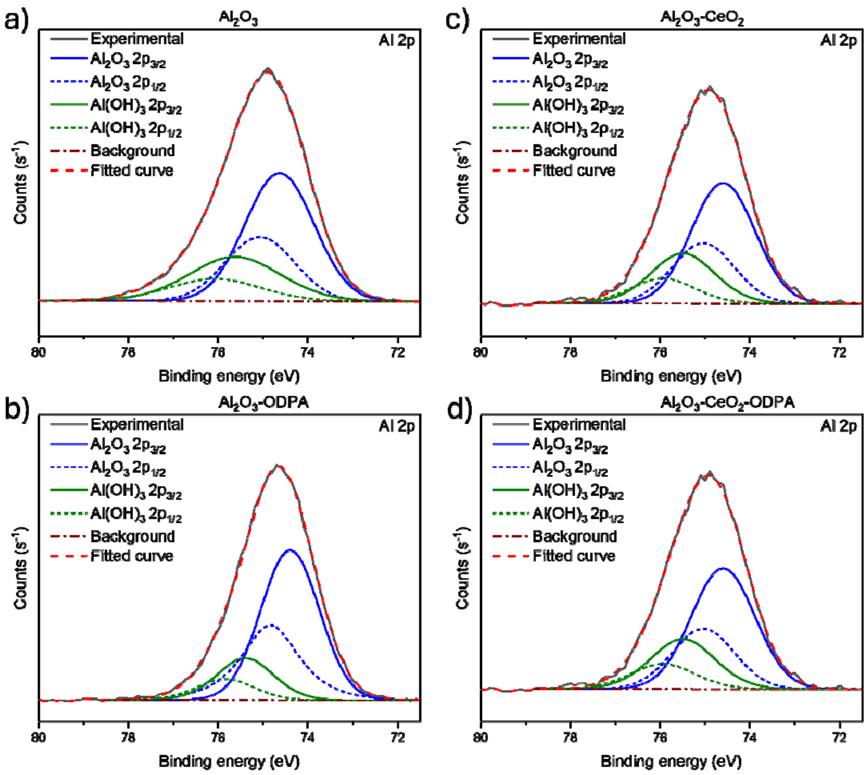


Figure S1. XPS high-resolution spectra for the Al 2p region from a) $Al_2O_3$, b) $Al_2O_3$-ODPA, c) $Al_2O_3$-$CeO_2$, and d) $Al_2O_3$-$CeO_2$-ODPA.

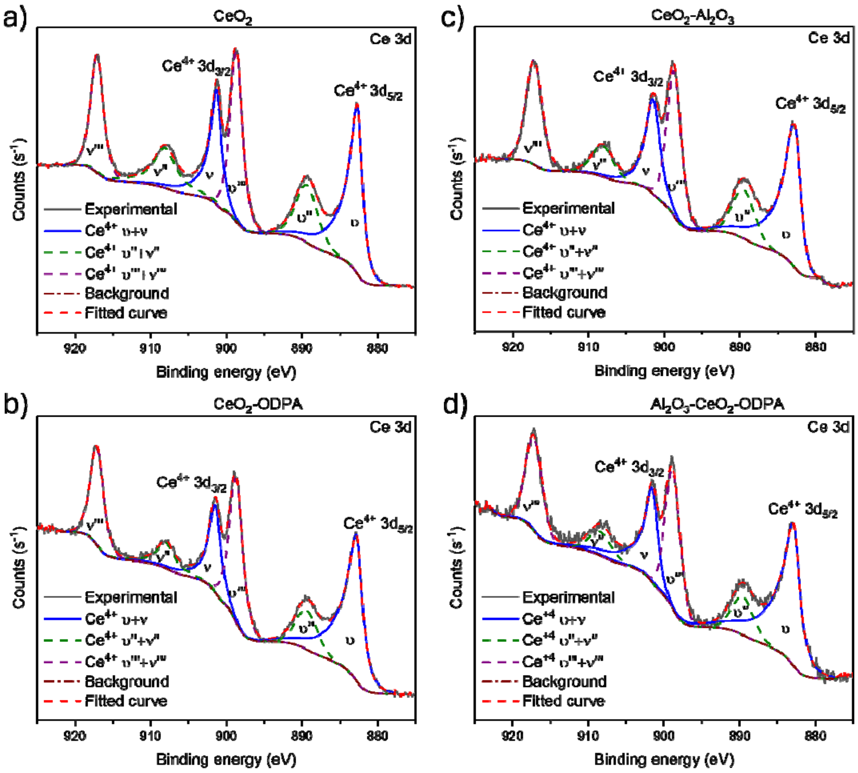


Figure S2. XPS high-resolution spectra for the Ce 3d region from a) $CeO_2$, b) $CeO_2$-ODPA, c) $Al_2O_3$-$CeO_2$, and d) $Al_2O_3$-$CeO_2$-ODPA.

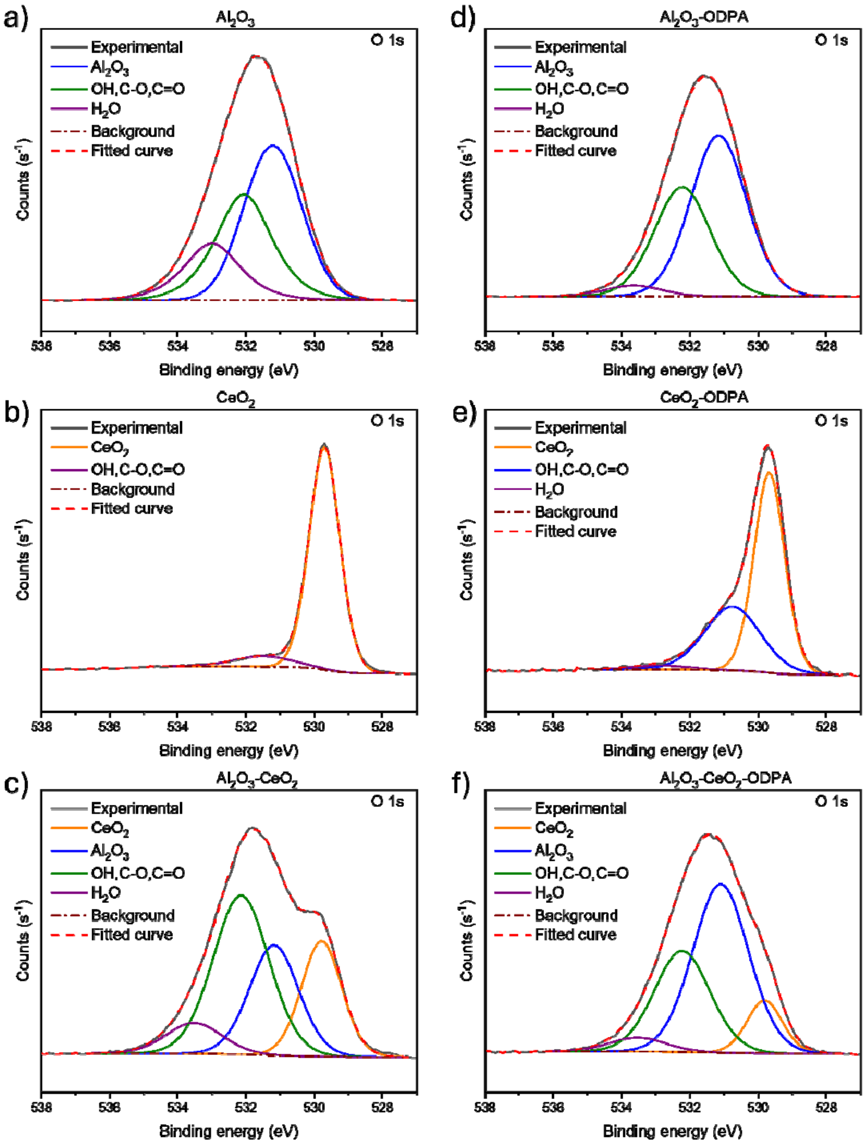


Figure S3. XPS high-resolution spectra for the O 1s region from a) $Al_2O_3$, b) $CeO_2$, c) $Al_2O_3$-$CeO_2$, d) $Al_2O_3$-ODPA, e) $CeO_2$-ODPA, and d) $Al_2O_3$-$CeO_2$-ODPA.
